# Comparison of the CAMI-NSTEMI and GRACE Risk Model for Predicting In-Hospital Mortality in Chinese Non-ST-Segment Elevation Myocardial Infarction Patients

**DOI:** 10.1155/2020/2469281

**Published:** 2020-07-24

**Authors:** Peng Wang, Hongliang Cong, Ying Zhang, Yujie Liu

**Affiliations:** ^1^Tianjin Medical University, Tianjin, China; ^2^Department of Cardiology, Tianjin Chest Hospital, Tianjin, China

## Abstract

**Introduction:**

The ability of risk models to predict in-hospital mortality and the influence on downstream therapeutic strategy has not been fully investigated in Chinese Non-ST-segment elevation myocardial infarction (NSTEMI) patients. Thus, we sought to validate and compare the performance of the Global Registry of Acute Coronary Events risk model (GRM) and China Acute Myocardial Infarction risk model (CRM) and investigate impacts of the two models on the selection of downstream therapeutic strategies among these patients.

**Methods:**

We identified 2587 consecutive patients with NSTEMI. The primary endpoint was in-hospital death. For each patient, the predicted mortality was calculated according to GRM and CRM, respectively. The area under the receiver operating characteristic curve (AUC), Hosmer–Lemeshow (H–L) test, and net reclassification improvement (NRI) were used to assess the performance of models.

**Results:**

In-hospital death occurred in 4.89% (126/2587) patients. Compared to GRM, CRM demonstrated a larger AUC (0.809 versus 0.752, *p* < 0.0001), less discrepancy between observed and predicted mortality (H–L *χ*^2^: 22.71 for GRM, *p*=0.0038 and 10.25 for CRM, *p*=0.2479), and positive NRI (0.3311, *p* < 0.0001), resulting in a significant change of downstream therapeutic strategy.

**Conclusion:**

In Chinese NSTEMI patients, the CRM provided a more accurate estimation for in-hospital mortality, and application of the CRM instead of the GRM changes the downstream therapeutic strategy remarkably.

## 1. Introduction

Non-ST-segment elevation myocardial infarction (NSTEMI) is a leading cause of mortality, morbidity, and hospitalization from cardiovascular disease both worldwide and in China, which has a major influence on health economies [1–4]. Unfortunately, lower rather than higher risk NSTEMI patients are more likely to receive more aggressive therapeutic strategies, which is the so-called risk-treatment paradox [5–7]. To diminish the impact of this paradox, the current guidelines consider the risk assessment by the Global Registry of Acute Coronary Events (GRACE) risk model as a fundamental component to select the most appropriate therapeutic strategy for NSTEMI patients [1–3]. However, several observations have suggested that the performance of the GRACE risk model (GRM) was unsatisfactory among Chinese NSTEMI patients [8, 9]. Recently, a novel risk model has been developed for the risk evaluation of NSTEMI patients [8] based on multicenter data from the China Acute Myocardial Infarction (CAMI) registry [10]. To date, the CAMI-NSTEMI risk model (CRM) has not been systematically validated in an external cohort. Moreover, it has not been fully demonstrated how these risk assessment models influenced clinical management. Thus, the present study tends to validate and compare the performance of GRM and CRM and investigate the impacts of the two proposed models on the selection of downstream therapeutic strategies in Chinese NSTEMI patients.

## 2. Materials and Methods

### 2.1. Study Participants

**Figure 1:**
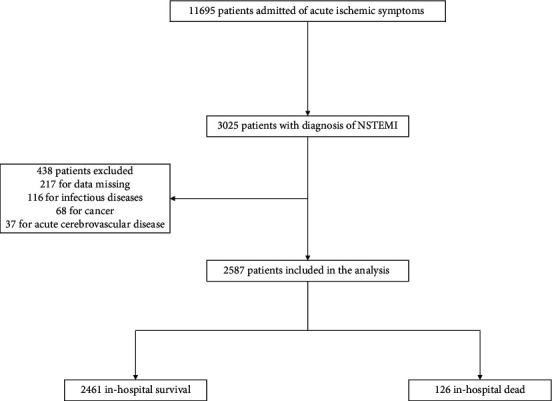
The flow diagram. NSTEMI: non-ST-segment elevation myocardial infarction.

The details of inclusion and exclusion criteria for the study participants are illustrated in Figure 1. According to the most recent guidelines, NSTEMI was defined as symptoms of ischemia and detection of an elevation of cardiac troponin values without new persistent ST-segment elevation [1–3]. Three thousand and twenty-five patients with a primary clinical diagnosis of NSTEMI were included. Then, the patients were excluded if they fulfilled one of the following criteria: data missing (217), acute or chronic infectious diseases (116), cancer (68), and acute cerebrovascular disease (37). Among the 217 patients excluded from the analysis for missing data, medical histories for 68% (147/217), results of blood test for 28% (61/217), and clinical presentations for 24% (52/217) were not available. At last, 2587 patients were included in this study and were divided into in-hospital survival and death groups. This observational study complied with the Declaration of Helsinki and was approved by the local Ethics Committees.

### 2.2. Data Collection and Definitions

The following variables were registered through review of the electronic medical record: age, sex, history of diabetes mellitus, hypertension, hyperlipidemia, smoking, previous revascularization, and previous myocardial infarction. The body mass index (BMI), Killip class, heart rate (HR), systolic blood pressure (SBP), creatinine (Cr), white blood cell count (WBC), cardiac arrest, and ST-segment depression on an 18-lead electrocardiogram at admission were also recorded. Hyperlipidemia was defined as a total cholesterol of at least 220 mg/dl, low-density lipoprotein cholesterol of at least 140 mg/dl, fasting triglycerides of at least 150 mm/dl, or receiving treatment with oral lipid-lowering agents. Diabetes was defined as fasting glucose levels over 7 mmol/l or treatment currently with diet, oral glucose-lowering agents, or insulin. The smoking status included current smoker, nonsmoker, and previous smoker (quit >6 month). The primary endpoint of this study was in-hospital death, defined as all-cause death during hospitalization.

### 2.3. Risk Assessment by the CRM and GRM

The GRM included 8 independent risk factors: age, Killip class, SBP, ST-segment depression, cardiac arrest, Cr, initial cardiac enzyme findings, and HR [11]. The initial cardiac enzyme findings were positive because all patients have been diagnosed with NSTEMI. According to the GRM and guideline recommendations, we classified patients into 3 risk groups: the low-, medium-, and high-risk group [1]. The CRM identified 11 independent predictors of in-hospital mortality: age, BMI, SBP, Killip class, cardiac arrest, ST-segment depression, Cr, WBC smoking status, previous MI, and previous percutaneous coronary intervention, and all patients were classified into 3 risk groups based on the CRM [8].

### 2.4. Statistical Analysis

All statistical analyses were carried out by MedCalc (version 15.2.2; MedCalc Software, Mariakerke, Belgium) and R (version 3.2.4; R Foundation for Statistical Computing, Vienna, Austria). A two-tailed *p* value less than 0.05 was considered statistically significant. Continuous variables were compared using Student's *t*-tests or Mann–Whitney *U*-tests as appropriate. Count variables were assessed using the *χ*^2^-test or Fisher's exact test as appropriate. This study conducting validation and comparison of multivariable prediction models strictly followed Transparent Reporting of a Multivariable Prediction Model for Individual Prognosis or Diagnosis (TRIPOD): the TRIPOD statement [12]. To validate and compare the predictive value of the GRM and CRM, we used three characteristics: discrimination, calibration, and classification [13]. Discrimination refers to how well the model differentiates those having an endpoint from those not having. The area under receiver-operator characteristic curve (AUC) was used to quantify the improvement in discrimination [14]. Calibration reflects the extent to which the values predicted by the model agree with the observed values. We used Hosmer–Lemeshow (H–L) tests which divided patients into ten groups according to deciles of mortality and calculated a chi-square statistic (H–L *χ*^2^) to assess calibration [15]. As the selection of downstream therapeutic strategy usually bases on the risk classification, we established a reclassification table to evaluate the net reclassification improvement (NRI), determining how correctly a model reclassifies patients into various risk categories compared with another [16].

## 3. Results

Baseline characteristics of the study cohort are listed in Table 1. Overall, hospital death occurred in 4.89% (126/2587) patients. Compared to the in-hospital survival group, the in-hospital dead group were older, had more male, lower BMI and SBP, and a higher prevalence of diabetes mellitus, hypertension, current smoker, cardiac arrest, high Killip class, and ST-segment depression. Also, HR, Cr, WBC, and troponin T were higher in the in-hospital dead group. All the differences were statistically significant (*p* < 0.05).

**Figure 2:**
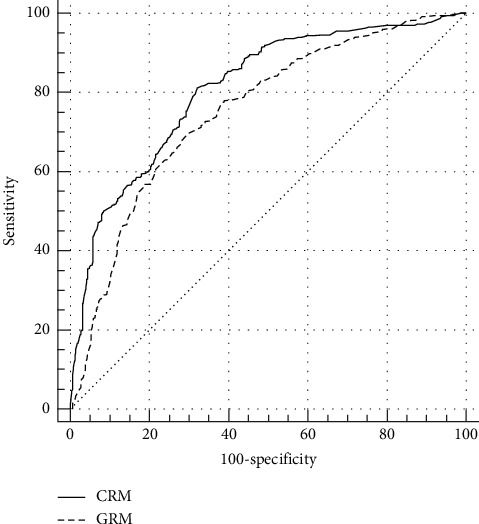
Comparison of 2 models by receiver operating characteristic curves. GRM: GRACE risk model; CRM: CAMI-NSTEMI risk model.

The receiver-operator characteristic curves of 2 models are exhibited in Figure 2. The AUC for the CRM (0.809, 95% confidence interval: 0.789 to 0.829, *p* < 0.0001) was significantly (*p* < 0.0001) larger than that for the GRM (0.752, 95% confidence interval: 0.729 to 0.774, *p* < 0.0001).

**Figure 3:**
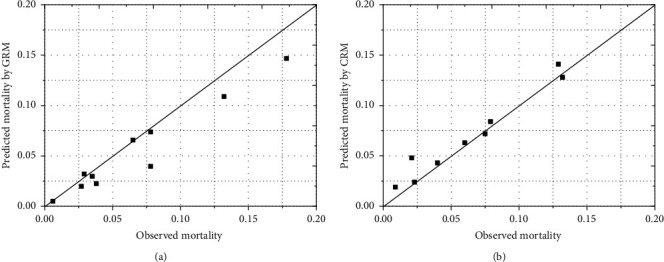
Predicted and observed mortality by deciles of predicted mortality. GRM: GRACE risk model; CRM: CAMI-NSTEMI risk model.

The predicted mortality was compared with the observed mortality in deciles of predicted mortality as illustrated in Figure 3. The GRM manifested a predominance of underestimation, resulting in a poor calibration (H–L *χ*^2^ = 22.71, *p*=0.0038). The differences between the observed and predicted mortality were not evident in H–L calibration plots for CRM, so the calibration of the CRM was good (H–L *χ*^2^ = 10.25, *p*=0.2479).

Table 2 shows the reclassification table comparing the CRM to GRM. For the 126 positive patients, compared to the GRM, the CRM correctly reclassified 23 from the medium- to high-risk category, 12 from low to high, and 6 from low to medium, but 2 from medium to low and 2 from high to medium. Of the 2461 negative patients, 174 were correctly reclassified to a lower risk category but 82 to a higher PTP category. As a result, compared to the GRM, the NRI for the CRM was 0.2937 in positive, 0.0374 in negative, and 0.3311 overall (*p* < 0.0001). In other words, the replacement of the GRM by CRM for every 2587/(23 + 12 + 6 + 174)≈13 patients would result in 1 correct reclassification.

## 4. Discussion

This observational analysis determined that the CRM provided a more effective prediction for in-hospital mortality in Chinese NESTEMI patients. Compared to the GRM, the CRM demonstrated a larger AUC, less discrepancy between the observed and predicted mortality, and a positive NRI. It was worth noting that the application of the CRM instead of the GRM may pronouncedly change the downstream therapeutic strategy in these NESTEMI patients.

Compared to ST-segment elevation myocardial infarction (STEMI) patients, NSTEMI patients present with more heterogeneous variation in ischemic risk and comorbidities so that the risk classification has been considered as a fundamental component to select the most appropriate therapeutic strategy for NSTEMI patients [1–3, 17]. However, in clinical practice the treatment-risk paradox is widespread [5–7], which is partly due to the suboptimal risk assessment underestimating the ischemic risk [18, 19], especially in China [4, 6, 20]. In conformity with this, according to the H–L calibration plots in the present study, the GRM dramatically underestimated the in-hospital mortality and classified nearly 40% in-hospital death into the low- or medium-risk category, which may cause underuse of invasive strategy for patients at high risk [1, 2]. Two reasons may potentially account for the unfavorable performance when applying GRM to the Chinese NSTEMI patients. First, GRM was developed in patients mainly from America, Europe and Australia twenty years ago [11]. There are significant differences in characteristics and management between these and contemporary Chinese patients [3, 11]. Second, the GRM was developed to assess the risk of patients with acute coronary syndrome, including STEMI, NSTEMI, and unstable angina pectoris [11].

To address the limitations mentioned above, the CRM was developed to predict in-hospital mortality, particularly for Chinese NSTEMI patients, and its diagnostic performance was superior to that of the GRM in two internal validation studies using data from CAMI registry [8, 9]. Our effort further extended this conclusion in an external validation cohort by indicating a larger AUC, positive NRI, and less disagreement between the observed and predicted mortality for the CRM. Moreover, the GRM classified 15% (19/126) in-hospital dead into the low-risk category, for which invasive strategy was not recommend according to current guidelines and 22% (28/126) into the medium-risk category, for which immediate (<2 h) and early (<24 h) invasive strategy were not recommend [1]. Using the CRM instead of the GRM would imply a significant change for downstream therapeutic strategy in the in-hospital dead: 87% (36/47) of these patients would be reclassified into the higher risk category, for which more aggressive strategies were recommended. Thus, application of the CRM instead of the GRM may have the potential to optimize the referral of aggressive intervention and lead to an evident diminution of the treatment-risk paradox in NSTEMI patients.

This study was subjected to the limitations of its single-center, retrospective, and observational design. The indication of clinical management and downstream therapeutic strategy were based on the individual physician decision. Thus, the strategic discordance of downstream management for NSTEMI patients should not be ignored, and the actual impact of applying the CRM was complicated. To further investigate the generalizability and reliability of CRM-guided therapeutic strategy, more pragmatic and cost-effective randomized control trials are needed, such as the UK GRACE Risk Score Intervention Study (UKGRIS) [21].

## 5. Conclusions

In Chinese NSTEMI patients, the CRM provided a more effective estimation for in-hospital mortality due to the improvement in discrimination, classification, and calibration compared to the GRM. The application of the CRM instead of the GRM could change the diagnostic strategy and the potential to optimize the referral of a more aggressive intervention.

## Figures and Tables

**Table 1:** Baseline characteristics of patients who died vs. survived.

Characteristic	Total (*n* = 2587)	In-hospital dead (*n* = 126)	In-hospital survival (*n* = 2461)	*p* value
Age	61.91 ± 10.97	72.84 ± 10.69	61.35 ± 9.74	<0.0001
Male	1290 (50)	84 (67)	1206 (49)	0.0001
BMI (kg/m^2^)	21.24 ± 3.91	21.14 ± 3.23	23.25 ± 3.74	<0.0001
Diabetes mellitus	540 (21)	48 (38)	492 (20)	<0.0001
Hypertension	1462 (57)	84 (67)	1378 (56)	0.0194
Hyperlipidemia	587 (23)	21 (17)	566 (23)	0.1146
Previous revascularization				
PCI	185 (7)	3 (3)	182 (7)	0.0628
CABG	45 (1.7)	1 (1)	44 (1.8)	0.5846
Previous MI	325 (12)	30 (24)	295 (12)	0.0027
Smoking status				<0.0001
Current smoker	855 (33)	18 (14)	837 (34)	
Previous smoker	393 (15)	24 (19)	369 (15)	
Nonsmoker	1339 (52)	84 (67)	1255 (51)	
HR (beats/min)	78.94 ± 29.47	90.01 ± 27.23	78.37 ± 21.14	<0.0001
SBP (mmHg)	127.70 ± 34.93	109.24 ± 33.73	128.65 ± 22.19	<0.0001
Cr (*μ*mol/L)	85.67 ± 98.64	144.64 ± 93.17	82.65 ± 63.38	<0.0001
WBC (10^9^/L)	8.33 ± 8.07	16.45 ± 7.66	7.91 ± 4.25	<0.0001
Troponin T (ug/l)	0.26 ± 2.24	1.47 ± 4.28	0.20 ± 1.37	<0.0001
Cardiac arrest	58 (2)	9 (7)	49 (2)	0.0007
Killip class				<0.0001
I	1598 (62)	23 (18)	1575 (64)	
II	463 (18)	20 (16)	443 (18)	
III	329 (13)	34 (27)	295 (12)	
IV	197 (7)	49 (39)	148 (6)	
ST-segment depression	1315 (51)	84 (67)	1231 (50)	0.0003

Values are presented as mean ± SD or *n* (%). BMI: body mass index; CABG: coronary artery bypass grafting; PCI: percutaneous coronary intervention; MI: myocardial infarction; HR: heart rate; SBP: systolic blood pressure; Cr: creatinine; WBC: white blood cell count.

**Table 2:** Reclassification table comparing the CRM to GRM.

	Risk category based on the CRM			Total	Reclassification^*∗*^		NRI^†^	*p*
	Low	Medium	High		Up	Down		
Risk category based on the GRM								
Negative patients					0.0333	0.0707	0.3311	<0.0001
Low	932	37	38	1007				
Medium	156	871	7	1034				
High	6	12	402	420				
Total	1094	920	447	2461				

Positive patients^‡^					0.3254	0.0317		
Low	1	6	12	19				
Medium	2	3	23	28				
High	0	2	77	79				
Total	3	11	112	126				

^*∗*^The classification of patients by the CRM was compared to that by the GRM. ^†^NRI = (P (Up|Positive)-P (Down|Positive)]-[ P (Up|Negative)-P (Down|Negative)). ^‡^A positive patient was defined as a patient who died in hospital. NRI: net reclassification improvement; GRM: GRACE risk model; CRM: CAMI-NSTEMI risk model.

## Data Availability

The data used to support the findings of this study are available from the corresponding author upon request.
