# Corrigendum to “Comparison of the CAMI-NSTEMI and GRACE Risk Model for Predicting In-Hospital Mortality in Chinese Non-ST-Segment Elevation Myocardial Infarction Patients”

**DOI:** 10.1155/2021/5137403

**Published:** 2021-02-10

**Authors:** Peng Wang, Hongliang Cong, Ying Zhang, Yujie Liu

**Affiliations:** ^1^Tianjin Medical University, Tianjin, China; ^2^Department of Cardiology, Tianjin Chest Hospital, Tianjin, China

In the article titled “Comparison of the CAMI-NSTEMI and GRACE Risk Model for Predicting In-Hospital Mortality in Chinese Non-ST-Segment Elevation Myocardial Infarction Patients” [1], the authors Hongliang Cong, Ying Zhang, and Yujie Liu were affiliated to the “Tianjin Medical University, Tianjin, China” which is incorrect. The correct affiliation for the authors Hongliang Cong, Ying Zhang, and Yujie Liu is “Department of Cardiology, Tianjin Chest Hospital, Tianjin, China.” The correct affiliations are also shown above.
